# Evaluation of risdiplam efficacy in 5q spinal muscular atrophy: A systematic comparison of electrophysiologic with clinical outcome measures

**DOI:** 10.1111/ene.16099

**Published:** 2023-10-12

**Authors:** Tobias Kessler, Georges Sam, Wolfgang Wick, Markus Weiler

**Affiliations:** ^1^ Department of Neurology Heidelberg University Hospital Heidelberg Germany; ^2^ Clinical Cooperation Unit Neurooncology, German Cancer Consortium (DKTK) German Cancer Research Center (DKFZ) Heidelberg Germany

**Keywords:** compound muscle action potential (CMAP), electrophysiology, neurodegeneration, risdiplam, spinal muscular atrophy (SMA)

## Abstract

**Background:**

To assess compound muscle action potential (CMAP) amplitudes as electrophysiologic markers in relation to clinical outcome in adult patients with 5q‐linked spinal muscular atrophy (SMA) before and during treatment with risdiplam.

**Methods:**

In this monocentric longitudinal cohort study, CMAP of 18 adult patients with SMA type 2 or 3 were assessed at baseline (*T*_0_) and after 10 months (*T*_10_) of risdiplam treatment. CMAP amplitudes of the median, ulnar, peroneal, and tibial nerves were compared with established clinical outcome scores, and with the course of disease before start of treatment.

**Results:**

During a pharmacotherapy‐naive pre‐treatment period of 328 ± 46 days, Revised Upper Limb Module (RULM) score and peroneal nerve CMAP amplitudes decreased, while CMAP of tibial and upper limb nerves remained unchanged. CMAP amplitudes positively correlated with clinical scores (Hammersmith Functional Motor Scale‐Expanded [HFMSE], RULM) at *T*_0_. During risdiplam treatment, HFMSE and Children's Hospital of Philadelphia Infant Test of Neuromuscular Disorders (CHOP INTEND) scores increased, paralleled by marked increase of CMAP amplitudes in both median nerves (*T*_10_–*T*_0_; right: Δ = 1.4 ± 1.4 mV, *p* = 0.0003; left: Δ = 1.3 ± 1.4 mV, *p* = 0.0007), but not in ulnar, peroneal, or tibial nerves. A robust increase of median nerve CMAP amplitudes correlated well with an increase in the HFMSE score (*T*_10_–*T*_0_). Median nerve CMAP amplitudes at *T*_0_ were associated with subsequent risdiplam‐related improvement of HFMSE and CHOP INTEND scores at *T*_10_.

**Conclusions:**

Median nerve CMAP amplitudes increase with risdiplam treatment in adult SMA patients, and should be further evaluated as potential easy‐to‐use electrophysiologic markers in assessing and monitoring clinical response to therapy.

## INTRODUCTION

5q‐linked spinal muscular atrophy (SMA) is an autosomal‐recessive motor neuron disease caused by mutations in the *survival of motor neuron (SMN)1* gene with retained function of a paralogous *SMN2* gene, resulting in a loss of functional SMN protein and consecutive degeneration of motor neurons in the ventral horn [1, 2]. Clinically, SMA is characterized by progressive skeletal muscle weakness and wasting [3, 4], producing a broad continuum of disease severity [5, 6].

New disease‐modifying drugs increasing SMN protein expression have been developed in the past decade and revolutionized SMA treatment. Most recently, risdiplam, an oral, well‐tolerated small molecule *SMN2* splicing modifier, was approved for the treatment of SMA patients, based on positive results from randomized placebo‐controlled clinical trials in patients with type 1, type 2, or non‐ambulant type 3 SMA, up to an age of 25 years [7, 8, 9]. The rise of several cost‐intensive innovative treatment options for SMA has led to a concomitant need of reliable biomarkers for meeting the challenges of therapeutic guidance and improved disease monitoring [10]. However, biomarkers appropriate to measure, monitor, or even predict the response to disease‐modifying drugs such as risdiplam are still lacking to date.

Alongside molecular and imaging markers, appliance‐based measures such as electrophysiologic recordings appear most promising to be developed as potential biomarkers for SMA in routine clinical practice [10]. The compound muscle action potential (CMAP) represents the summated action potentials of all stimulated motor endplates of a skeletal muscle upon supramaximal electrostimulation, and thus reflects the functional status of the involved motor unit pool. Previous data derived from therapy‐naïve patients demonstrate that maximum CMAP amplitudes are distinct among SMA types and correlate with *SMN2* copy numbers, age, and clinical function [11]. Therefore, CMAP recordings might be useful to objectively monitor clinical courses in SMA patients and assess responses to available pharmacotherapies including risdiplam.

The aim of the present study was to prospectively investigate longitudinal CMAP recordings as a potential biomarker in adult SMA patients before and during treatment with risdiplam, and correlate these findings with well‐established clinical outcome scores.

## METHODS

### Standard protocol approvals, registration, and patient consents

This exploratory, monocentric, longitudinal cohort study was approved by the institutional ethics board (University of Heidelberg; S‐554/2018), and all participants gave written informed consent. The study conforms with The Code of Ethics (Declaration of Helsinki, World Medical Association, 2013).

### Study design

**FIGURE 1:**
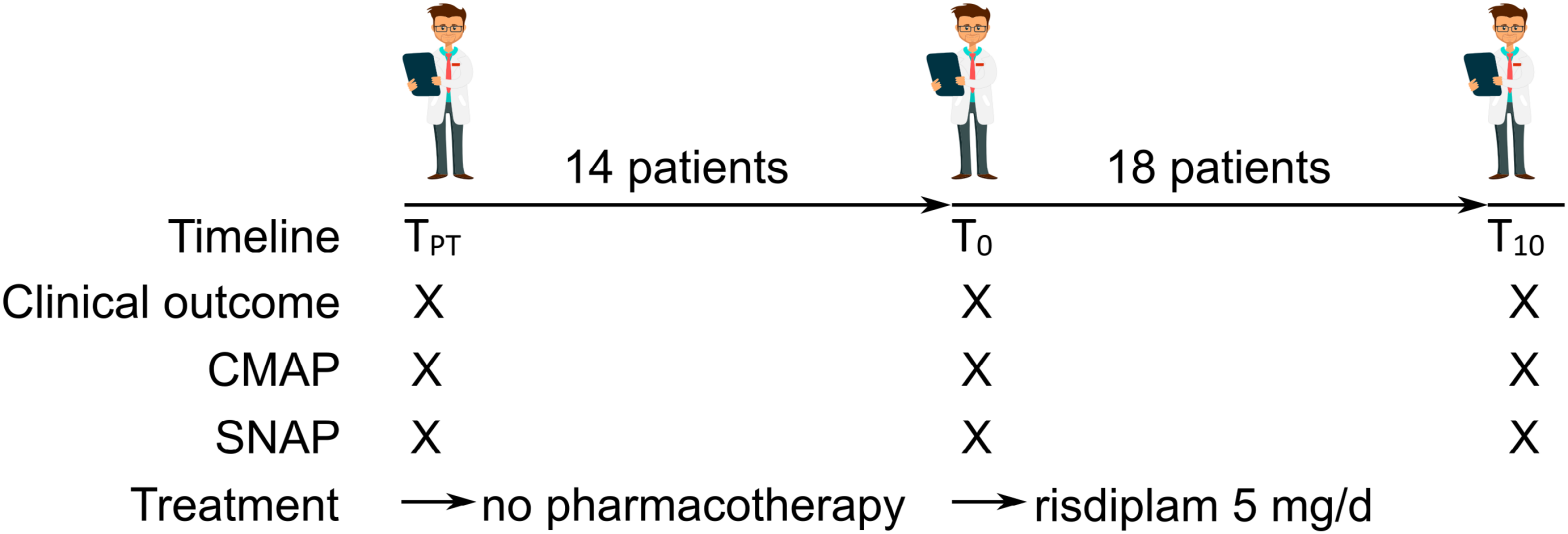
Flowchart of the study. CMAP, compound muscle action potential; SNAP, sensory nerve action potential; *T*_PT_, pre‐treatment; *T*_0_, start of treatment; *T*_10_, 10 months of treatment.

This study included an observational part in treatment‐naïve SMA patients and a subsequent longitudinal analysis of physiotherapeutic assessments during 10 months of treatment with risdiplam. A detailed flowchart of study assessments is given in Figure 1. The study was not pre‐registered and there was no sample size calculation in advance. The only inclusion criterion was a genetically confirmed 5q‐linked SMA. Exclusion criteria were age <18 years, pregnancy, and pre‐treatment with any *SMN*‐specific medication. Patients with impaired respiratory function or swallowing difficulties were allowed for accrual, as long as a safe oral application of risdiplam was deemed possible.

Treatment with risdiplam was at the decision of a board‐certified neurologist with more than 12 years of specialized experience in neuromuscular diseases including SMA (M.W.). SMA types were assigned clinically based on the age at symptom onset and achievement of motor milestones [12]. Patients were additionally assigned based on new classification to “Non‐Sitter”, “Sitter”, and “Walker”. No randomization or blinding was performed in this study. At baseline (*T*_0_), a detailed medical history was taken in all patients, including comprehensive neurologic, physiotherapeutic, and electrophysiologic examinations were performed. Risdiplam was administered orally at a dose of 5 mg per day. Comprehensive clinical and electrophysiologic follow‐up examinations as detailed above were performed 10 months after baseline for comparison (*T*_10_).

To assess the course of disease prior to pharmacotherapy, data from the same clinical and electrophysiologic examinations before baseline (*T*_PT,_ PT for “pre‐treatment”) were assessed from the clinical records of all study patients when available.

### Clinical outcome measures

Severity of clinical symptoms was assessed by physiotherapists with longstanding experience in SMA (Heidi Rochau‐Trumpp; Guido Stocker; professional experience >20 years, >8 years with SMA patients), applying the Hammersmith Functional Motor Scale‐Expanded (HFMSE) for the evaluation of gross motor functions, and the Revised Upper Limb Module (RULM) as the most robust scale for assessment of upper limb function in SMA [13, 14]. In patients unable to sit without assistance for at least 10 seconds, the Children's Hospital of Philadelphia Infant Test of Neuromuscular Disorders (CHOP INTEND) was applied as an additional validated rating system to assess gross motor functions in more severely affected patients [15, 16]. Physiotherapists were blinded for electrophysiologic findings. Increases of ≥3 points in the HFMSE score and/or ≥2 points in the RULM score were defined as cut‐off values for the definition of objective clinical improvement [13, 14].

### Nerve conduction studies

All participants of the study underwent routine motor and sensory nerve conduction studies performed by the same technician (G.S.) using the same Dantec™ Keypoint® G4 workstation according to standardized protocols at all timepoints throughout the entire study. Details are given in the supplementary methods.

### Statistical analysis

Statistical analysis was performed in R (version 4.2.2, RRID:SCR_001905) and Microsoft Excel 2016 (Microsoft Corporation, RRID:SCR_016137). Graphics were plotted with custom‐adapted scripts based on the R packages “ggplot2” (version: 3.3.6, RRID:SCR_014601) and “ComplexHeatmap” (version: 2.12.0, RRID:SCR_017270). Normal distributions were assessed with the Shapiro–Wilk test before parametric testing. Mean and standard error of the mean (SEM) values are given for agglomerated data. The Wilcoxon test was used for paired comparison. Correction for multiple testing was performed using the Benjamini–Hochberg method in R. Receiver operator characteristics (ROC) were calculated with the R package “pROC” (version 1.18.4). The Youden index was used to define the optimal cut‐off value. Customized R scripts are available upon reasonable request.

## RESULTS

### Patient demographics

A total of 25 pharmacotherapy‐naïve, non‐pediatric SMA patients (12 male, 13 female, mean age 37.0 ± 2.6 years, range 18–69 years) were consecutively enrolled at the Department of Neurology at Heidelberg University Hospital. All patients had a homozygous deletion of exons 7 and 8 of the *SMN1* gene. Of these 25 patients, 18 patients (8 male, 10 female, mean age 37.8 ± 3.2 years, range 18–69 years) started first‐line treatment with risdiplam between October 2020 and March 2022 at our institution (Table 1). Six patients were treated within the compassionate use program before risdiplam was clinically approved in Germany in March 2021 [17]. Of these 18 patients, 9 patients were classified as non‐sitters, 7 patients as sitters, and 2 patients as walkers. Seven patients had SMA type 2, and 11 patients had SMA type 3 (8 patients with SMA type 3a and 3 patients with SMA type 3b). Patients with SMA type 1 or 4 were not available at the study center during the accrual period. Mean age was 37.8 ± 3.2 years (range 18–69 years, 10 female and 8 male). Mean duration of clinical symptoms at study entry was 35.3 ± 3.1 years (range 17–66 years). At study entry, the mean HFMSE score was 6.9 ± 3.7, the mean CHOP INTEND score was 23.2 ± 1.6, and the mean RULM score was 14.4 ± 2.4. The study thus included preponderantly severely impaired patients, largely matching the clinical status of previously reported SMA cohorts receiving first‐line treatment with risdiplam (Table S1). The clinical and electrophysiologic baseline assessment (*T*_0_) was less than 1 week before the start of treatment with risdiplam. The second assessment (*T*_10_) was 10 months afterwards (mean 296 ± 2.9 days, range 277–336 days; Figure 1).

**TABLE 1:** Clinical and genetic characteristics of patients with spinal muscular atrophy treated with risdiplam (*n* = 18 patients).

Patient	SMA type	SMA class	Age (years)	Gender (M/F)	Duration of symptoms (years)	*SMN2* copies (*n*)	Spondylodesis	HFMSE (0–66) *T*_0_	CHOP INTEND (0–64) *T*_0_	RULM (0–37) *T*_0_
1	2	N	20	M	20	3	Yes	0	12	3
2	3a	S	26	M	24	4	Yes	4	31	14
3	3a	S	26	M	24	3	Yes	2	25	16
4	2	S	25	M	25	3	Yes	2	30	17
5	3a	S	22	F	20	3	Yes	0	27	15
6	3b	S	41	M	32	4	No	7	N/A	17
7	3b	W	38	F	30	4	No	53	N/A	37
8	2	N	42	F	42	3	Yes	0	23	5
9	3a	N	38	F	36	4	Yes	0	24	12
10	3b	S	69	F	66	4	No	4	24	14
11	2	N	40	M	40	3	Yes	0	25	8
12	3a	N	42	M	40	3	Yes	0	22	14
13	2	N	34	F	34	4	No	0	24	16
14	3a	N	38	F	37	3	Yes	0	27	10
15	2	N	42	M	42	3	No	0	10	0
16	2	N	60	F	59	3	No	0	16	2
17	3a	S	49	F	47	4	No	6	N/A	23
18	3a	W	18	F	17	3	No	46	N/A	37
Mean	N/A	N/A	37.8	N/A	35.3	3.39	N/A	6.89	23.2	14.4
SEM	N/A	N/A	3.18	N/A	3.12	0.12	N/A	3.70	1.57	2.40

Abbreviations: CHOP INTEND, Children's Hospital of Philadelphia Infant Test of Neuromuscular Disorders; F, female; HFMSE, Hammersmith Functional Motor Scale‐Expanded; M, male; N, non‐sitter; N/A, not applicable; RULM, Revised Upper Limb Module; S, sitter; SEM, standard error of the mean; SMA, spinal muscular atrophy; W, walker.

For the assessment of the course of disease prior to treatment, clinical and electrophysiologic data prior to the start of pharmacotherapy were available for 7/18 SMA patients treated with risdiplam. For 11 patients in the risdiplam treatment group, sufficient data before baseline were not available. Seven patients with available pre‐treatment data were not treated with risdiplam but treated with nusinersen thereafter; however, effects of nusinersen treatment were not analyzed in this study. The pre‐treatment cohort of patients (*n* = 14, Table S2) had a shorter median duration of symptoms and tended to be less severely impaired than patients in the risdiplam‐treated group whereas the latter was not statistically significant (Table S3). The mean time between the first (*T*_PT_) and second (*T*_0_) clinical and electrophysiologic assessment before therapy was 328 ± 46 days (range 91–652 days). This time interval approximated the ~10 months (296 ± 2.9 days) between the assessments before (*T*_0_) and after therapy (*T*_10_) in the risdiplam‐treated patient group.

### Clinical outcome before and during treatment with risdiplam

During the pharmacotherapy‐free time period, a reduction of the RULM score was observed (*p* = 0.042; Figure S1b), while there were no changes in HFMSE scores (Figure S1a). CHOP INTEND data were not available for the time before initiation of pharmacotherapy. After initiation of treatment with risdiplam, clinical improvement defined as increase of ≥3 points in the HFMSE and/or ≥2 points in the RULM score (see Methods) was achieved in 7/18 patients (38.9%). Improvement in the clinical scores was associated with a higher number of *SMN2* gene copies (Table S4). Mean increase in the HFMSE score (ΔHFMSE *T*_10_–*T*_0_) was 1.17 ± 0.39 (*p* = 0.014; Figure S2a). Similarly, CHOP INTEND scores increased during the 10 months of treatment (*p* = 0.002; Figure S2b), whereas there were no changes in the RULM score (*p* = 0.089; Figure S2c).

### CMAP before and during treatment with risdiplam

**FIGURE 2:**
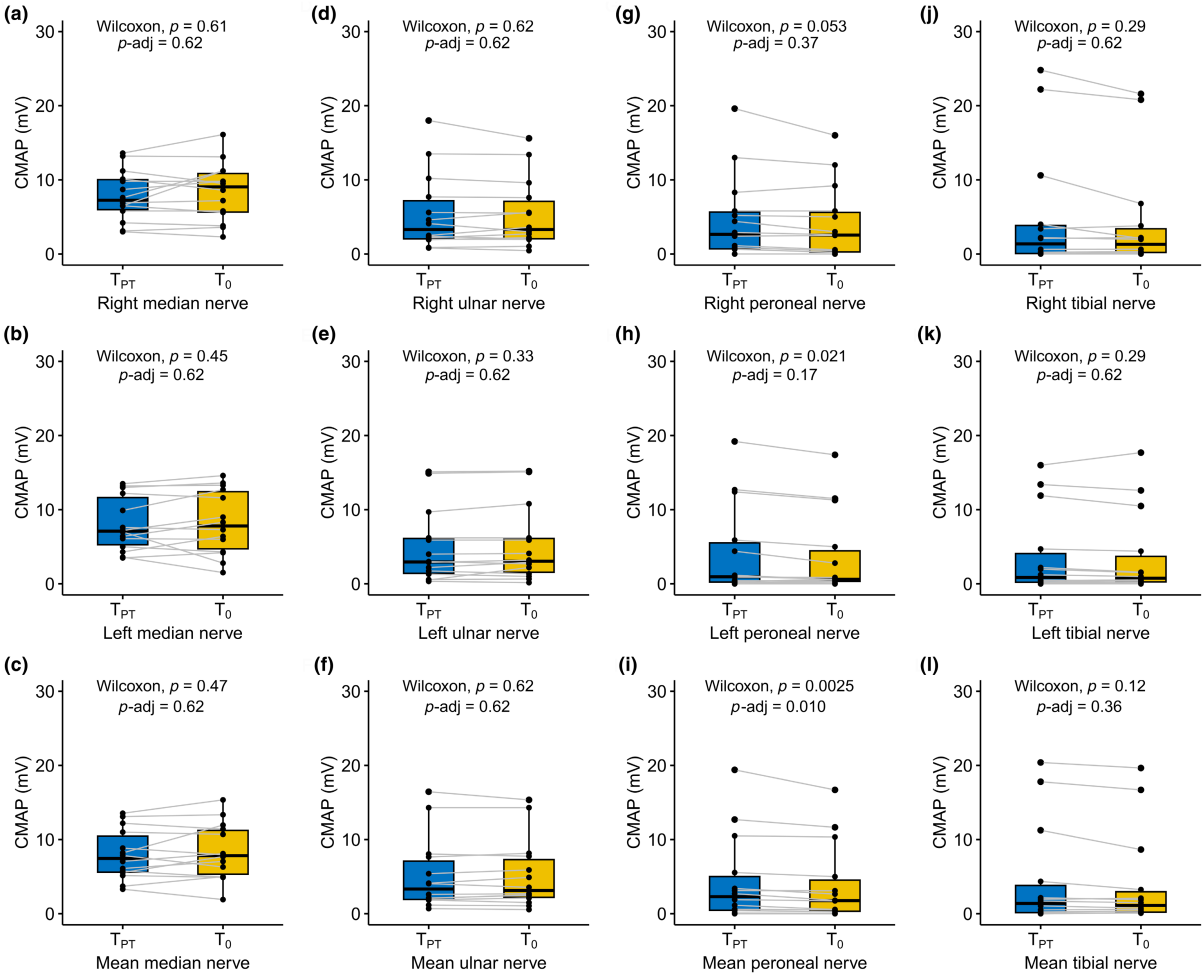
Compound muscle action potential (CMAP) amplitudes in pharmacotherapy‐naive adult patients with spinal muscular atrophy during the pre‐treatment period. CMAP amplitudes before treatment (*T*_PT,_ mean 328 ± 46 days) and at the time of treatment start (*T*_0_, *n* = 14 patients). (a–c) Median nerve; (d–f) ulnar nerve; (g–i) peroneal nerve; (j–l) tibial nerve. Measurements are shown as right side, left side, and mean values from both sides. Boxplots show mean and first to third quartiles. Whisker extends from the hinge to the largest value no further than 1.5 × interquartile range from the hinge. *P*‐values are calculated with the Wilcoxon test, raw *p*‐values and *p*‐values adjusted for multiple testing (*p*‐adj, Benjamini–Hochberg method) are given. *T*_PT_, pre‐treatment; *T*_0_, start of treatment.

Before initiation of pharmacotherapy, mean CMAP amplitudes of the median, ulnar, and tibial nerves did not change (Figure 2a–f,j–l). There was a trend towards a reduction of CMAP amplitudes of the right (*p* = 0.053, *p*‐adj = 0.37; Figure 2g) and left peroneal nerve (*p* = 0.021, *p*‐adj = 0.17; Figure 2h), though not significant after adjustment for multiple testing. Assessment of mean values of both sides revealed robust reduction of the peroneal nerve CMAP amplitude (*p* = 0.0025, *p*‐adj = 0.01; Figure 2i).

**FIGURE 3:**
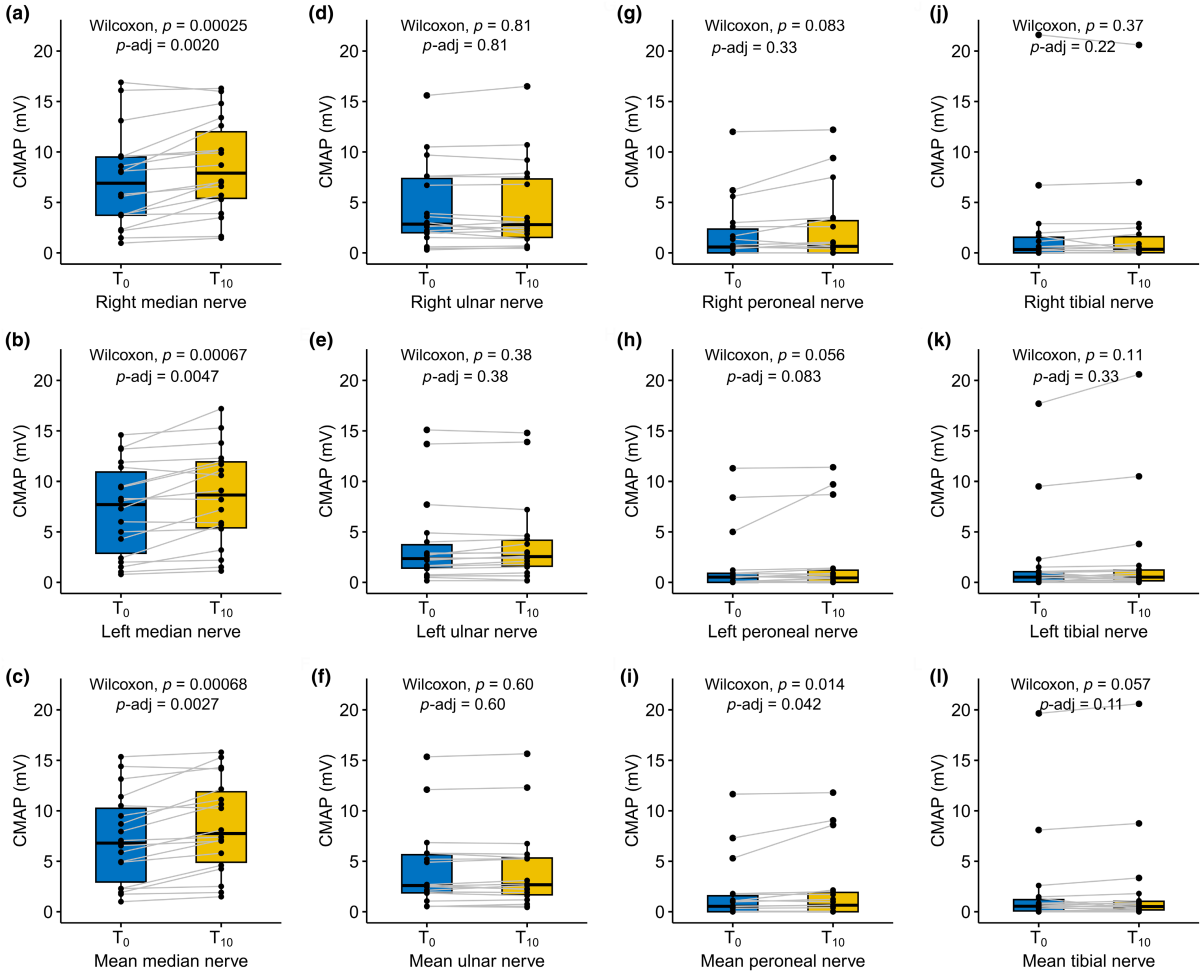
Changes of compound muscle action potential (CMAP) amplitudes in adult patients with spinal muscular atrophy during treatment with risdiplam. CMAP amplitudes at start (*T*_0_) and after 10 months of treatment with risdiplam (*T*_10_) (*n* = 18 patients). (a–c) Median nerve; (d–f) ulnar nerve; (g–i) peroneal nerve; (j–l) tibial nerve. Measurements are shown as right side, left side, and mean values from both sides. Boxplots show mean and first to third quartiles. Whisker extends from the hinge to the largest value no further than 1.5 × interquartile range from the hinge. *P*‐values are calculated with the Wilcoxon test, raw *p*‐values and *p*‐values adjusted for multiple testing (*p*‐adj, Benjamini–Hochberg method) are given. *T*_0_, start of treatment; *T*_10_, 10 months of treatment.

The mean CMAP amplitude of the median nerve at *T*_0_ was 7.2 ± 1.1 mV (range 1.0–16.9 mV) for the right side and 7.2 ± 1.1 mV (range 0.8–14.6 mV) for the left side. CMAP of the median nerve increased on the right side (Δ = 1.4 ± 1.4 mV, *p* = 0.0003, *p*‐adj = 0.002; Figure 3a), the left side (Δ = 1.3 ± 1.4 mV, *p* = 0.0007, *p*‐adj = 0.0047; Figure 3b), and the mean of both sides during treatment with risdiplam (*p* = 0.00068, *p*‐adj = 0.0027; Figure 3c). The mean CMAP amplitude of the ulnar nerve at *T*_0_ was 4.6 ± 1.0 mV (range 0.5–15.6 mV) for the right side and 3.7 ± 1.0 mV (range 0.2–15.1 mV) for the left side. There were no relevant changes in CMAP of the right and left ulnar nerves or in mean values from both sides in response to risdiplam treatment (Figure 3d–f). The mean baseline CMAP amplitudes of the lower limb nerves were generally low in our cohort: right peroneal nerve: 1.9 ± 0.7 mV (range 0–12.0 mV), left peroneal nerve: 1.7 ± 0.8 mV (range 0–11.3 mV), right tibial nerve: 2.1 ± 1.2 mV (range 0–21.6 mV), and left tibial nerve: 2.1 ± 1.1 mV (range 0–17.7 mV). During treatment with risdiplam, there were no changes of CMAP amplitudes of the peroneal or tibial nerves, neither on both sides separately nor the mean (Figure 3g–l). A comparison with normal CMAP reference values is given in Table S5.

Sensory nerve action potential (SNAP) amplitudes of the median and ulnar nerves were normal on both sides, while SNAP amplitudes of the sural nerves were generally low. SNAP amplitudes did not change during treatment with risdiplam (Figure S3a–f).

### Correlation of CMAP with clinical scores

**FIGURE 4:**
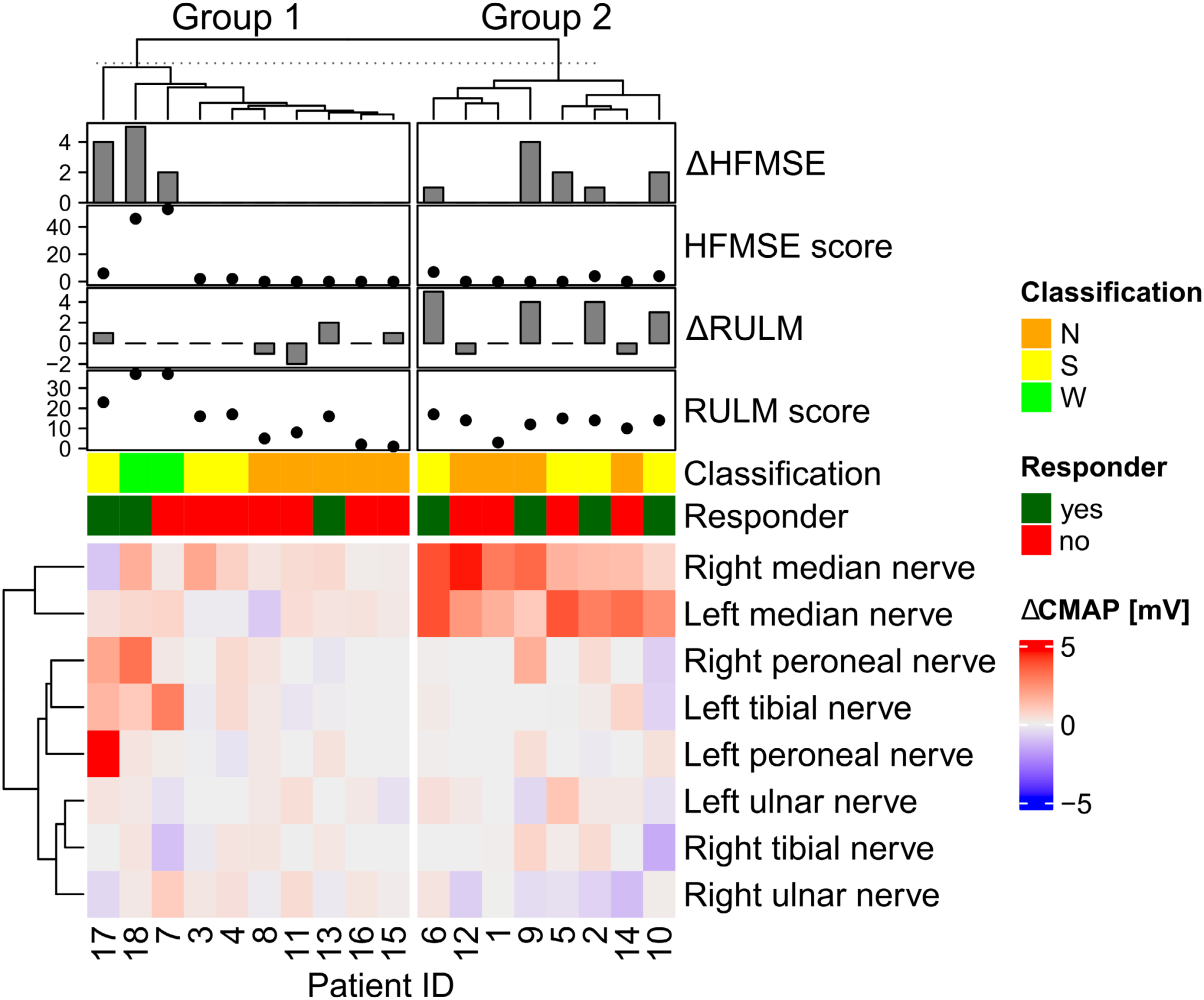
Correlation of compound muscle action potential (CMAP) amplitudes with clinical scores. Heatmap showing *k*‐means clustering of CMAP amplitudes of the four measured peripheral nerves on both sides annotated with HFMSE score, change in HFMSE score (ΔHFMSE; *T*_10_–*T*_0_), RULM score, change in RULM score (ΔRULM; *T*_10_–*T*_0_), ambulatory status, and responder status according to definition in the Methods (increase [*T*_10_–*T*_0_] in HFMSE ≥3 and/or RULM ≥2, *n* = 18 patients). Group 1 consists of patients without median nerve CMAP changes, group 2 consists of patients with an increase in median nerve CMAP amplitudes in response to risdiplam. HFMSE, Hammersmith Functional Motor Scale‐Expanded; N, non‐sitter; RULM, Revised Upper Limb Module; S, sitter; W, walker. Bottom annotation numbers represent patient identification numbers according to Table 1.

HFMSE and RULM scores at *T*_0_ were positively correlated with CMAP amplitudes in all four examined peripheral nerves on both sides, whereas there was no correlation between CMAP amplitudes and the CHOP INTEND score (Figures S4–S6). The CMAP amplitudes best reflected the HFMSE score, in particular in the lower limbs (*R* = 0.77–0.93, *p* = 6.1 × 10^−6^–2.5 × 10^−11^; Figure S4). Changes in CMAP amplitudes in response to treatment with risdiplam were then compared with changes in clinical scores. Clustering of CMAP amplitude changes of the eight examined nerves revealed two groups: group 1 without changes, and group 2 showing a marked increase in the CMAP amplitude in one or both median nerves (Figure 4).

Of the seven patients who clinically responded to risdiplam treatment, four were in the group that showed a strong increase in median nerve CMAP amplitudes. Two patients with clinical response but without an increase in median nerve CMAP amplitudes and subsequent group 1 assignment had an increase in CMAP amplitudes of the peroneal nerve on the left and/or right side (Patients 17 and 18). There was one patient who formally fulfilled the clinical criteria of a responder (Patient 13) whose CMAP amplitudes did not increase under risdiplam treatment. However, the assignment of this patient as a responder was based on a two‐point increase in the RULM score, without any improvement in the HFMSE score (Figure 4).

### Correlation of CMAP at baseline with subsequent response to risdiplam treatment

**FIGURE 5:**
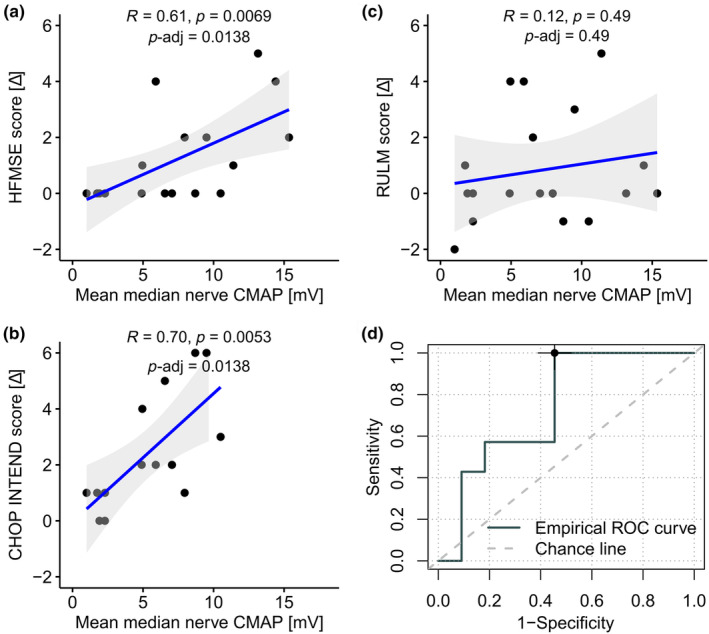
Changes in clinical scores based on baseline compound muscle action potential (CMAP) values and response prediction. (a–c) Baseline mean median nerve CMAP amplitudes at *T*_0_ compared with the change in different clinical scores during the 10 months of treatment with risdiplam (*T*_10_–*T*_0_). (a) HFMSE score (*n* = 18 patients); (b) CHOP INTEND score (*n* = 14 patients); (c) RULM score (*n* = 18 patients); (d) Receiver operating characteristic (ROC) shows sensitivity and specificity for different cut‐offs based on mean median CMAP amplitude to predict therapy response. Black dot indicates optimal cut‐off point. CHOP INTEND, Children's Hospital of Philadelphia Infant Test of Neuromuscular Disorders; HFMSE, Hammersmith Functional Motor Scale‐Expanded; RULM, Revised Upper Limb Module.

Finally, the applicability of CMAP measurements at *T*_0_ to predict clinical improvement after 10 months of treatment with risdiplam was assessed. Based on our previous results, we focused on median nerve CMAP for all further assessments. Mean baseline median nerve CMAP amplitudes (*T*_0_) correlated well with the subsequent risdiplam‐related improvement of HFMSE (mean *R* = 0.61, *p* = 0.0069, *p*‐adj = 0.138; Figure 5a) and CHOP INTEND scores (mean *R* = 0.7, *p* = 0.0053, *p*‐adj = 0.138; Figure 5b). However, no correlation was found between mean median nerve CMAP amplitudes at *T*_0_ and subsequent improvement in RULM score in response to risdiplam treatment (Figure 5c). Assessments for each side separately revealed the same associations (Figure S7). Based on the positive correlation with clinical scores, we identified cut‐off values for median nerve CMAP to predict response to therapy using ROC curves. A mean median nerve CMAP value of 5.0 mV predicted clinical response to risdiplam treatment with 100% sensitivity and 54% specificity (Table 2; Figure 5d).

**TABLE 2:** Comparison of median nerve compound muscle action potential (mean of both sides) for response prediction in receiver operating characteristic analysis (*n* = 18 patients).

Response prediction	Cut‐off (mV)	AUC	ACC	SENS	SPEC	FSCR
Median nerve (right side)	3.7	0.71	0.61	1.00	0.36	0.67
Median nerve (left side)	4.3	0.73	0.66	1.00	0.45	0.70
Median nerve (mean)	5.0	0.74	0.72	1.00	0.54	0.73

*Note*: Data are given for the right and left median nerve separately as well as the mean value of both sides. Optimal (Youden index) point matches SENS = 1.00 in all three analyses.

Abbreviations: ACC, accuracy; AUC, area under the curve; SENS, sensitivity; SPEC, specificity; FSCR, F‐score.

## DISCUSSION

The present longitudinal cohort study was conducted in adult patients with SMA type 2 or 3 before and during treatment with risdiplam. We observed declining peroneal nerve and unaltered tibial, median, and ulnar nerve CMAP amplitudes alongside slightly decreased or stable clinical scores within a median observation interval of more than 10 months before initiation of treatment, approximating the natural course of disease. In contrast, the first 10 months of treatment with risdiplam led to a robust increase in CMAP amplitudes of both median nerves. Therapy‐related recovery of motor neuron function within the given time frame of 10 months seems more likely in the upper limbs because most study participants were non‐ambulatory and had highly degenerated lower limb motor neuron function and muscle wasting.

The positive response of the median nerve CMAP in comparison to the non‐responsive ulnar nerve CMAP might be explained by a specific wasting pattern of hand muscles described in SMA patients. Based on motor unit number index (MUNIX) recordings [18], the thenar muscles including the abductor pollicis brevis muscle innervated by the median nerve were shown to be relatively preserved compared with hypothenar muscles, a denervation pattern sometimes referred to as ‘reversed split hand’ [19]. Accordingly, the less affected median nerve could be more responsive to a risdiplam‐mediated reconstitution of motor neuron function resulting in elevated CMAP amplitudes specifically in this arm nerve. This is of relevance as nerve conduction studies performed in SMA patients both within clinical registries and routine clinical practice commonly examine the ulnar and peroneal nerves only [20]. However, our results suggest that CMAP recordings of the median nerve could be more meaningful for assessing and monitoring treatment effects, and that this should be considered in future clinical investigations.

This recovery was paralleled by improvement of well‐established clinical scores (HFMSE, CHOP INTEND). However, there were no significant differences in the RULM score in response to risdiplam. The reason for this may be speculative, but the overall number of patients in our cohort was not powered for such an analysis, and potential improvements on muscular fatigue may be better reflected by a physiotherapeutic test of whole‐body functions such as the HFMSE, rather than the RULM. Notably, applying stringent cut‐off values for clinical improvement, more patients clinically improved according to RULM (≥2 points) than to HFMSE (≥3 points) cut‐offs.

CMAP amplitudes in all four examined peripheral nerves on both sides positively correlated with clinically relevant physiotherapeutic assessment scores (HFMSE, RULM) at *T*_0_, which, taken together, comprehensively reflect the patient's physical state. The missing correlation with the CHOP INTEND may be explained by the fact that this score was only determined in a lower number of severely affected patients, mostly non‐sitters, all with highly reduced CMAP amplitudes. The low SNAP amplitudes of sural nerves are not uncommon in severely impaired SMA patients and may be largely attributed to ankle and/or lower leg edema as a consequence of chronic immobility and muscle wasting.

CMAP clustering analysis revealed two groups of risdiplam‐related response patterns: group 1 without median nerve CMAP changes, and group 2 with an increase in median nerve CMAP amplitudes. Together, these data emphasize that a clinically relevant treatment response to risdiplam is linked to an induction of CMAP amplitudes.

Moreover, median nerve CMAP amplitudes at *T*_0_ strongly correlated with a subsequent increase in HFMSE and CHOP INTEND scores thus indicating that the extent of a therapeutic response to risdiplam at *T*_10_ was greater in physically fitter patients. This result is in line with findings from a previous multicenter observational study on nusinersen treatment demonstrating that, independent of age, adult SMA patients with more preserved motor functions benefit from a disease‐modifying treatment more than clinically more disabled patients [21].

The present study provides electrophysiologic data on the effects of risdiplam in SMA. A major strength of our work is that therapy‐related findings are compared with the course of disease prior to pharmacotherapy in preponderantly the same patients over a comparably long time interval. Our results show that median nerve CMAP amplitudes increase under risdiplam treatment leading to reported and clinically measurable improvements in adult SMA patients, an effect that has not been observed in the natural course of the disease [11].

However, our study has limitations. The low number of patients assessed limited the ability to define a confirmed median nerve CMAP cut‐off value that would allow prediction of response to risdiplam. ROC curves demonstrate that all responders to risdiplam treatment had a normal mean median nerve CMAP of ≥5 mV at baseline, thus enabling mean median nerve CMAP as a potential candidate marker for response with high sensitivity and moderate specificity. Further research is needed to assess this threshold. In our cohort, all except for one clinical responder to risdiplam had four *SMN2* copies. Consequently, it is not possible to assess the independence of CMAP cut‐offs from *SMN2* copy numbers regarding response prediction. A recently initiated comprehensive longitudinal prospective cohort study to assess peripheral motor function with electrophysiologic techniques in patients with SMA could apply and test median nerve CMAP as a biomarker in a larger patient cohort [22].

Our dataset was intentionally confined to the effects of risdiplam and does not provide head‐to‐head comparisons with nusinersen. To avoid confounding, we did not include patients who had previously switched from nusinersen to risdiplam. Furthermore, clinical characteristics are hererogeneously distributed and show a preponderance of severely diseased non‐sitters and sitters, restricting the applicability of our findings to this patient population. However, the patients studied here resemble the previously reported risdiplam‐treated patient cohorts [9, 23, 24]. The cohort assessing the course of disease prior to pharmacotherapy is imbalanced to some degree regarding symptom duration and a tendency towards less severely affected patients, possibly biasing comparative analyses. Finally, the consecutive accrual may be prone to selection bias.

Various putative biomarker sources for SMA have been under investigation, including clinical, electrophysiologic, imaging, molecular, and digital biomarkers [10, 25, 26, 27]. Other than imaging technologies [28, 29, 30, 31, 32], electrophysiologic recordings, including CMAP measurements, motor unit number estimation (MUNE), and MUNIX, assess the functional status of the motor unit pool and are therefore important in the diagnostic process and monitoring of disease progression in motor neuron disorders. While CMAP measures the electrical output of a muscle, MUNE estimates the number of innervating motor neurons [26]. MUNE was proposed as a surrogate marker to predict benefit from treatment with nusinersen in milder affected adult SMA patients [33]. However, in contrast to routine motor nerve conduction studies, MUNE is more time‐consuming and established only at a few specialized centers, while CMAP recordings are instead ubiquitously available at every neurological department. They are time‐ and cost‐efficient, non‐invasive, easy to perform, and do not require cooperation or repositioning of the mostly severely physically handicapped (electric) wheelchair‐dependent patients. CMAP was shown to improve in pediatric patients treated with nusinersen [34] or onasemnogene abeparvovec [35], but therapy‐related data on CMAP in adult SMA patients have been lacking to date.

Conclusively, we demonstrate with the present study that treatment with risdiplam increases median nerve CMAP amplitudes in ambulatory and non‐ambulatory adult patients with SMA, indicating clinical improvement. Median nerve CMAP should be further assessed in larger cohorts as a potential electrophysiologic biomarker that is widely available, patient‐friendly, and easy‐to‐use in the application of disease‐modifying pharmacotherapies for SMA.

## AUTHOR CONTRIBUTIONS

**Tobias Kessler**: Investigation; Conceptualization; Writing—original draft; Methodology; Validation; Visualization; Writing—review & editing; Formal analysis; Resources; Data curation; Software. **Georges Sam**: Investigation; Methodology; Writing—review & editing; Data curation. **Wolfgang Wick**: Writing—review & editing; Formal analysis; Data curation; Resources. **Markus Weiler**: Conceptualization; Investigation; Funding acquisition; Writing—original draft; Methodology; Validation; Writing—review & editing; Formal analysis; Project administration; Supervision; Resources; Data curation; Visualization; Software.

## FUNDING INFORMATION

The study was supported in part by the 2nd Felix Jerusalem Award of the German Society for Muscle Diseases granted to M.W. in 2021.

## CONFLICT OF INTEREST STATEMENT

T.K. reports no disclosures. G.S. reports no disclosures. W.W. reports honoraria for consultation or non‐financial clinical trial support from Apogenix, Bayer, Merck Sharp & Dome, AstraZeneca, Merck Serono, Novartis, Roche, and Mundipharma, with compensation paid to the University Clinic. M.W. reports consultant honoraria from Biogen and Roche, and speaker honoraria and travel support for conference attendance from Biogen.

## Supporting information


Data S1.

## Data Availability

The data that support the findings of this study are available from the corresponding author upon reasonable request.
