# Supplementary material for: Evaluation of risdiplam efficacy in 5q spinal muscular atrophy: A systematic comparison of electrophysiologic with clinical outcome measures
Source: Eur J Neurol. 2023 Oct 12;31(1):e16099. doi: 10.1111/ene.16099 (PMC11235981; doi:10.1111/ene.16099)
Supplement: Supplementary file 1 — Data S1. [file ENE-31-e16099-s001.pdf]

Supplementary Material

Evaluation of risdiplam efficacy in 5q spinal muscular atrophy: a systematic comparison of electrophysiologic with clinical outcome measures

Tobias Kessler, MD,[1,2] Georges Sam, PhD,[1] Wolfgang Wick, MD,[1,2] and Markus Weiler, MD[1]

[1]Department of Neurology, Heidelberg University Hospital, Heidelberg, Germany
[2]Clinical Cooperation Unit Neurooncology, German Cancer Consortium (DKTK), German Cancer Research Center (DKFZ), Heidelberg, Germany

Corresponding author
Markus Weiler, MD
Department of Neurology
Heidelberg University Hospital
Im Neuenheimer Feld 400
D-69120 Heidelberg
Germany
Phone: +49 6221 567504
Fax: +49 6221 565461
Email: markus.weiler@med.uni-heidelberg.de

Supplementary Methods

Nerve conduction studies

All participants of the study underwent routine motor and sensory nerve conduction studies performed by the same technician (G.S.) using the same Dantec™ Keypoint® G4 workstation at all timepoints throughout the entire study. G.S. had more than 15 years of professional experience in electrodiagnostics and was blinded for clinical outcome results during the course of the entire study. Motor nerve conduction studies (NCS) were performed in the median nerve (recording of abductor pollicis brevis muscle), ulnar nerve (recording of abductor digiti minimi muscle), peroneal nerve (recording of extensor digitorum brevis muscle), and tibial nerve (recording of abductor hallucis muscle), each on both sides. CMAP were recorded according to the muscle belly-tendon principle applying a supramaximal stimulating current (stimulus widths: 0.2 ms) to the peripheral nerve at the wrist or ankle in a defined distance from an adhesive recording electrode placed onto the skin overlying the respective target muscle (median: 7 cm; ulnar: 5 cm; peroneal: 7 cm; tibial: 8 cm). In detail, current was applied at least 20% over the maximum measured CMAP amplitude to ensure validity of the measurement. Total CMAP amplitudes were measured from negative to positive peak. Normal CMAP values were defined for the median and tibial nerves ≥ 5.0 mV and for the ulnar and peroneal nerve ≥ 4.0 mV. For the present analysis, only CMAP amplitudes generated from the respective distal nerve electrostimulation site were assessed, i.e., from wrist for the median and ulnar nerves, and from ankle for the peroneal and tibial nerves. Sensory nerve action potentials (SNAP) were measured antidromically using adhesive electrodes for the median, ulnar, and sural nerves, each on both sides. SNAP amplitudes were measured from baseline to negative peak. Normal SNAP values for the median nerve were ≥ 7.0 µV, for the ulnar nerve ≥ 5.6 µV, and for the sural nerve ≥ 5.0 µV. Skin temperature was controlled at a minimum of 32°C.

Supplementary Tables

Table S1. Baseline clinical characteristics of studies on adult SMA patients treated with risdiplam.

Study	N	HFMSE [mean ± SD]	RULM [mean ± SD]	Cohort
Kessler et al. (present study)	18	6.9 ± 3.7	14.4 ± 2.4	Adult patients (> 18 years)
McClusky et al. 2022 [1]	5	N/A	8.6 ± 2.7	Adult patients (> 18 years)
Ñungo Garzón et al. 2023 [2]	6	N/A	3.2 ± 4.6	Non-sitters above (> 16 years)
Mercuri et al. 2022 [3]	120	16.1 ± 12.5	19.7 ± 7.2	Adult and pediatric patients

Table S2. Baseline characteristics of patients in the pre-treatment cohort (n = 14 patients).

**Patient No.**	**SMA type**	**SMA class**	**Age [years]**	**Gender [m/f]**	**Duration of symptoms [years]**	***SMN2* copies [n]**	**Spondylodesis**	**HFMSE [0-66] T_{PT}**	**RULM [0-37] T_{PT}**
1	2	N	20	m	20	3	yes	0	6
2	3a	S	26	m	24	4	yes	4	19
3	3a	S	26	m	24	3	yes	2	16
4	2	S	25	m	25	3	yes	5	15
5	3a	S	22	f	20	3	yes	4	16
6	3b	S	41	m	32	4	no	9	20
7	3b	W	38	f	30	4	no	49	37
19	3a	S	31	m	29	3	no	8	18
20	3a	S	41	m	39	4	no	5	18
21	3a	N	30	f	30	3	yes	4	13
22	3b	W	57	m	39	4	no	62	37
23	3a	S	21	m	20	4	no	17	24
24	3a	S	23	f	19	3	no	21	22
25	3b	S	40	f	29	3	no	7	22
**Mean**	N/A	N/A	32.0	N/A	27.1	3.43	N/A	14.1	20.2
**SEM**	N/A	N/A	2.84	N/A	1.77	0.14	N/A	4.97	2.24

N, Non-sitter; S, Sitter; W, walker; N/A, not applicable; m, male; f, female. Patients 1-7 are identical with patients 1-7 in Table 1.

Table S3. Comparison of clinical characteristics between the pre-treatment and the risdiplam-treated cohort.

	**Pre-treatment**	**Risdiplam**	**p-value**
Patients (n)	14	18	N/A
SMA type<br>- 2<br>- 3a<br>- 3b	 2 (14.3%)<br>8 (57.1%)<br>4 (28.5%)	 7 (38.9%)<br>8 (44.4%)<br>3 (16.7%)	 0.37
SMA class<br>- Non-sitter<br>- Sitter<br>- Walker	 2 (14.3%)<br>10 (71.4%)<br>2 (14.3%)	 9 (50.0%)<br>7 (38.9%)<br>2 (11.1%)	 0.12
Age in years [mean ± SEM]	32.0 ± 2.8	37.8 ± 3.2	0.19
Gender<br>- female<br>- male	 5 (35.7%)<br>9 (64.3%)	 10 (55.6%)<br>8 (44.4%)	 0.49
Duration of symptoms in years [mean ± SEM]	27.1 ± 1.8	35.3 ± 3.1	0.032
SMN2 copies [mean ± SEM]	3.4 ± 0.1	3.4 ± 0.1	0.83
HFMSE [mean ± SEM]	14.1 ± 5.0	6.9 ± 3.7	0.25
CHOP INTEND [mean ± SEM]	N/A	23.2 ± 1.6	N/A
RULM [mean ± SEM]	20.2 ± 2.2	14.4 ± 2.4	0.19

Table S4. Relation between number of *SMN2* copies and responder status (n = 18 patients).

	**Non-responder**	**Responder**	**p-value**
3 *SMN2* copies	10	1	0.0025
4 *SMN2* copies	1	6	

Table S5. Patients with normal and reduced CMAP amplitude at T_0 and T_{10} on both sides (n = 18 patients).

	**T_0 right**	**T_0 left**	**T_{10} right**	**T_{10} left**
Median nerve				
≥ 5.0 mV	11 (61.1%)	12 (66.7%)	14 (77.8%)	14 (77.8%)
< 5.0 mV	7 (38.9%)	6 (33.3%)	4 (22.2%)	4 (22.2%)
Ulnar nerve				
≥ 4.0 mV	6 (33.3%)	5 (27.8%)	6 (33.3%)	5 (27.8%)
< 4.0 mV	12 (66.7%)	13 (72.2%)	12 (66.7%)	13 (72.2%)
Peroneal nerve				
≥ 4.0 mV	3 (16.7%)	3 (16.7%)	3 (16.7%)	3 (16.7%)
< 4.0 mV	15 (83.3%)	15 (83.3%)	15 (83.3%)	15 (83.3%)
Tibial nerve				
≥ 5.0 mV	2 (11.1%)	2 (11.1%)	2 (11.1%)	2 (11.1%)
< 5.0 mV	16 (88.9%)	16 (88.9%)	16 (88.9%)	16 (88.9%)

Supplementary Figures

Figure S1

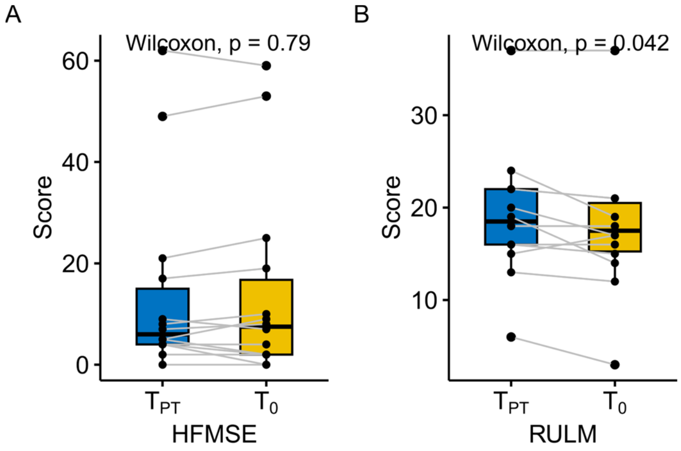

Figure S1. Clinical scores in pharmacotherapy-naive adult patients with SMA during the pre-treatment period. Clinical scores before treatment ($T_{PT,}$ mean 328 ± 46 days) and at the time of treatment start (T_0). A, HFMSE; B, RULM (n = 14 patients each). Boxplots show mean and first to third quartiles. Whisker extends from the hinge to the largest value no further than 1.5x inter-quartile range from the hinge.

Figure S2

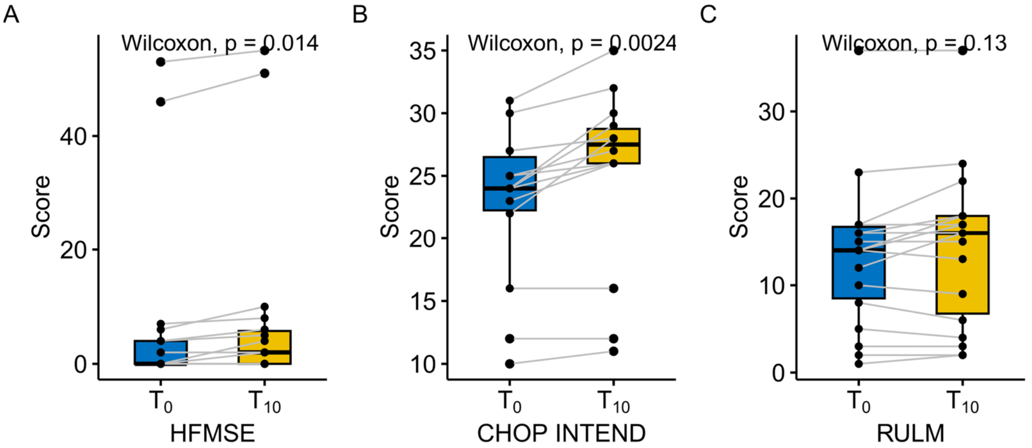

Figure S2. Clinical scores assessed at start of treatment (T_0) and after ten months of treatment (T_{10}) with risdiplam. A, HFMSE (n = 18 patients); B, CHOP INTEND (n = 14 patients); C, RULM (n = 18 patients). Boxplots show mean and first to third quartiles. Whisker extends from the hinge to the largest value no further than 1.5x inter-quartile range from the hinge.

Figure S3

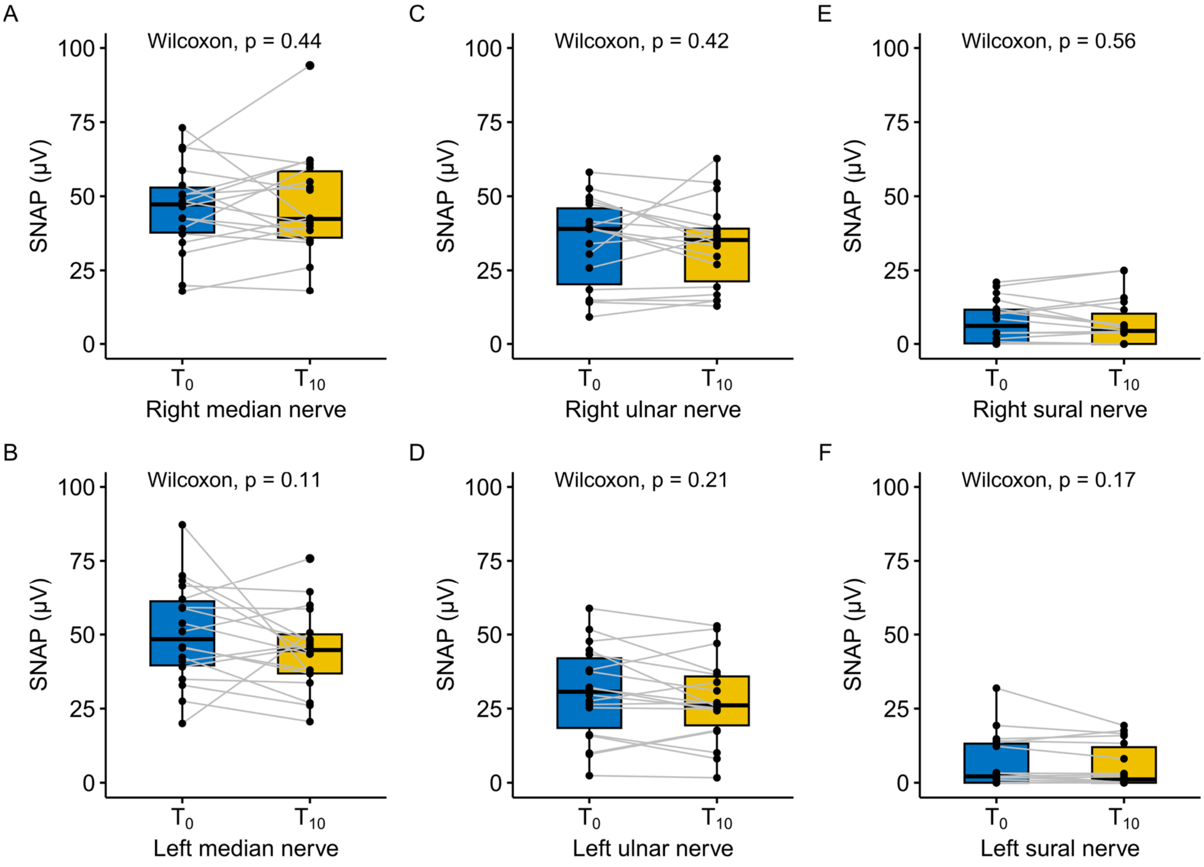

Figure S3. Changes of sensory nerve action potential (SNAP) amplitudes in adult patients with SMA during treatment with risdiplam. SNAP amplitudes at start (T_0) and after ten months of treatment (T_{10}) (n = 18 patients). A-B, median nerve; C-D, ulnar nerve; E-F, sural nerve. Boxplots show mean and first to third quartiles. Whisker extends from the hinge to the largest value no further than 1.5x inter-quartile range from the hinge.

Figure S4

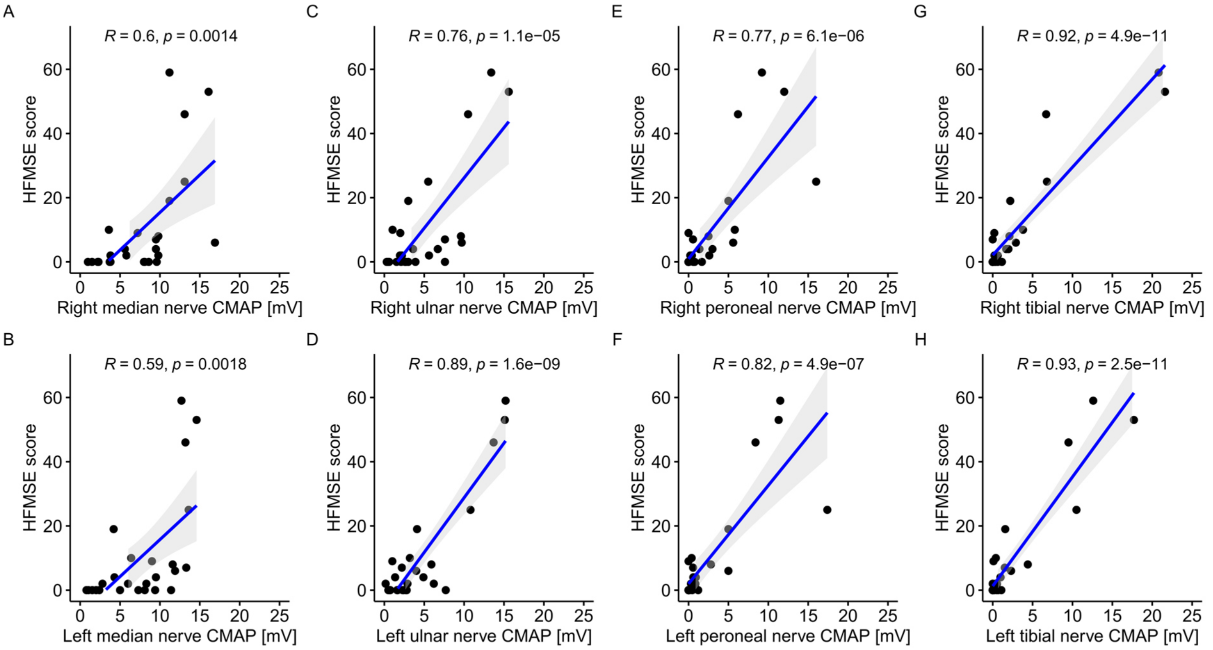

Figure S4. Correlations between baseline CMAP amplitudes and HFMSE scores. A-B, median nerve; C-D, ulnar nerve; E-F, peroneal nerve; G-H, tibial nerve (n = 25 patients each).

Figure S5

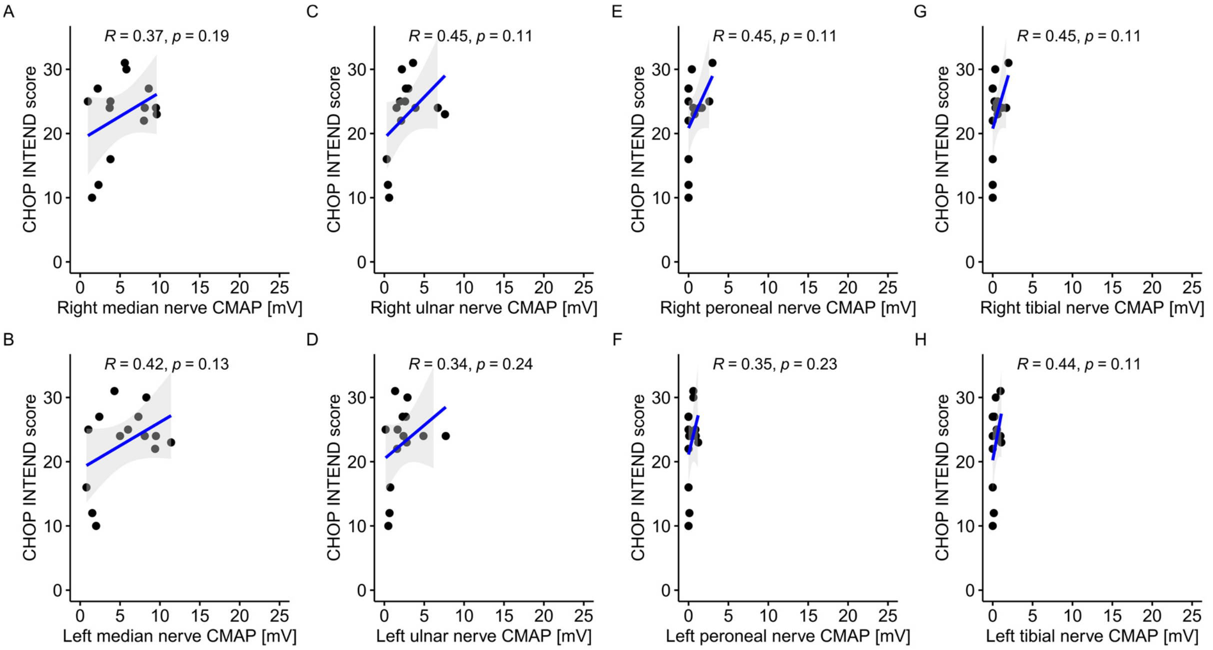

Figure S5. Correlations between baseline CMAP amplitudes and CHOP INTEND scores. A-B, median nerve; C-D, ulnar nerve; E-F, peroneal nerve; G-H, tibial nerve (n = 14 patients each).

Figure S6

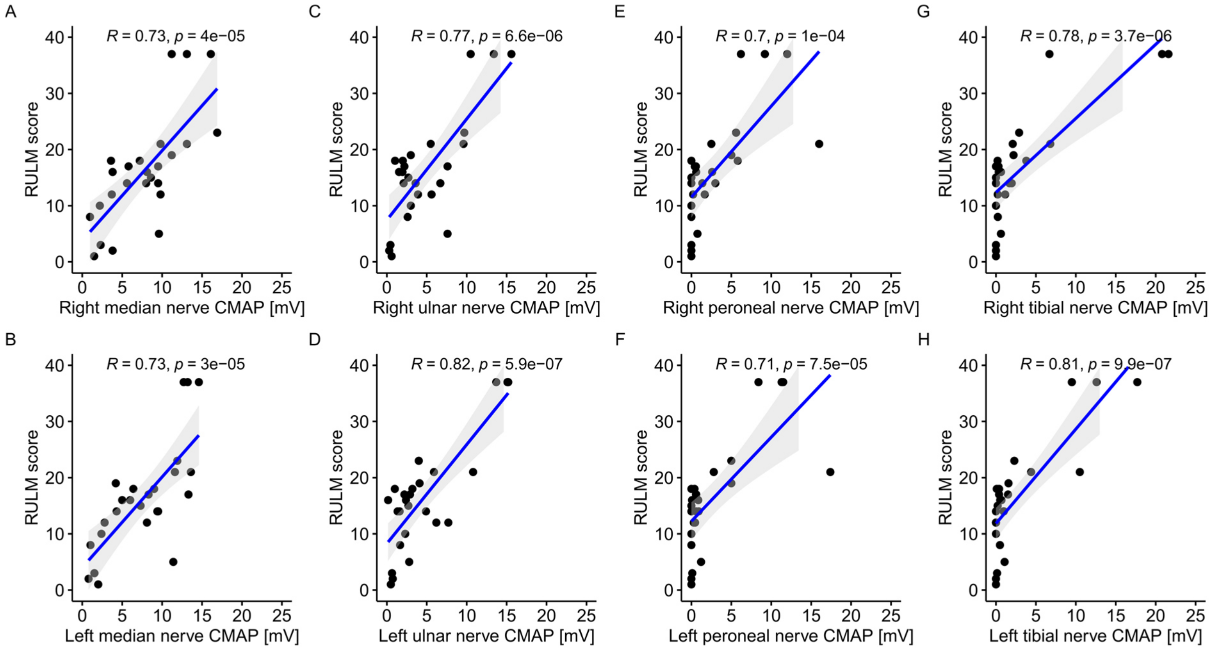

Figure S6. Correlations between baseline CMAP amplitudes and RULM scores. A-B, median nerve; C-D, ulnar nerve; E-F, peroneal nerve; G-H, tibial nerve (n = 25 patients each).

Figure S7

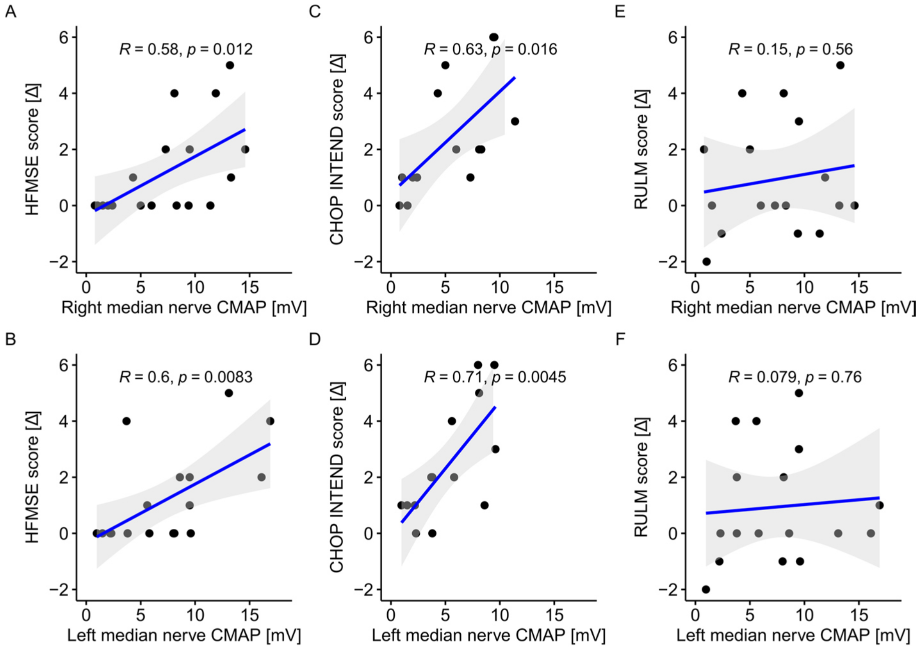

Figure S7. Change in clinical scores in response to risdiplam treatment correlated with baseline median nerve CMAP amplitudes. Baseline median nerve CMAP amplitudes at T_0 compared with the change in different clinical scores during ten months of treatment with risdiplam (T_{10}-T_0). A-B, HFMSE (n = 18 patients); C-D, CHOP INTEND (n = 14 patients); E-F, RULM (n = 18 patients).

Supplementary References

1. McCluskey G, Lamb S, Mason S, et al. Risdiplam for the treatment of adults with spinal muscular atrophy: Experience of the Northern Ireland neuromuscular service. *Muscle Nerve*. 2023;67(2):157-161.
2. Nungo Garzon NC, Pitarch Castellano I, Sevilla T, Vazquez-Costa JF. Risdiplam in non-sitter patients aged 16 years and older with 5q spinal muscular atrophy. *Muscle Nerve*. 2023;67(5):407-411.
3. Mercuri E, Deconinck N, Mazzone ES, et al. Safety and efficacy of once-daily risdiplam in type 2 and non-ambulant type 3 spinal muscular atrophy (SUNFISH part 2): a phase 3, double-blind, randomised, placebo-controlled trial. *Lancet Neurol*. 2022;21(1):42-52.
